# *In vitro* evaluation of the probiotic potential of *Lactobacillus* isolated from native swine manure

**DOI:** 10.14202/vetworld.2021.1133-1142

**Published:** 2021-05-11

**Authors:** Chiraprapha Tuyarum, Aporn Songsang, Monthon Lertworapreecha

**Affiliations:** 1 Microbiology Program, Department of Biology, Faculty of Science, Thaksin University, Phatthalung, 93210, Thailand; 2 Faculty of Technology and Community Development, Thaksin University, Phatthalung, 93210, Thailand

**Keywords:** *Lactobacillus* spp, native swine manure, probiotic properties

## Abstract

**Background and Aim:**

Using antimicrobials as a feed additive in swine production is prohibited because it is a major cause of the emergence of antimicrobial-resistant bacteria. Probiotics such as *Lactobacillus* spp. are an attractive alternative to reduce antimicrobial resistance and promote swine growth. This study aimed to evaluate the *in vitro* probiotic properties of *Lactobacillus* isolated from indigenous swine manure.

**Materials and Methods:**

A total of 30 fecal samples from healthy individual indigenous pigs were collected and isolated on de Man, Rogosa, and Sharpe agar. The preliminary screen identified candidates with antibacterial activity against six pathogens and >50% survival and tolerance to acid (pH 3.0) and 1% bile salt. Isolates that passed the initial screen will be tested for other probiotic properties.

**Results:**

Of the 314 isolates from 30 pig manure samples, 17 isolates satisfied all initial conditions for probiotic properties. Each isolate has unique, distinctive properties. Isolates B4, B5, B8, B17, B87, and B144 formed thick biofilms, whereas isolates B5, B8, and 27 adhered well to the intestinal wall and exhibited strong autoaggregation properties. Isolate B4 aggregated with Enterohemorrhagic *Escherichia coli* and Enteropathogenic *E. coli*. Tests in pH-adjusted cell-free medium indicated that the antibacterial activity resulted from bacterial acidification rather than bacteriocin formation. Sequence analysis (*16S rRNA*) revealed 16 of the isolates were *Lactobacillus plantarum*, and only one isolate was *Lactobacillus salivarius*.

**Conclusion:**

We isolated 17 *Lactobacillus* from swine manure and demonstrated that their probiotic properties might be useful as a probiotic cocktail for swine feed.

## Introduction

Many countries have prohibited antimicrobial feed additives in animal husbandry [1]. Although subtherapeutic antimicrobial doses stimulate livestock growth, their adverse effects encourage pathogenic bacteria in the digestive tract to adapt and become resistant to those antimicrobials. Moreover, resistant bacteria can transfer resistance genes to other bacteria in the environment, rapidly resulting in widespread resistance. The consequence of the ban on antimicrobials in animal feed is an increased incidence of gastrointestinal tract infections. A Danish study indicated that the incidence of *Escherichia coli* and *Lawsonia intracellularis* infection in pigs significantly increased in post-weaned pigs after the ban of antimicrobials. Morbidity due to gastrointestinal tract infections in post-weaned pigs also increased 600% [2].

The use of *Lactobacillus* spp. to replace antimicrobials has become an attractive alternative [3]. Numerous studies confirm that dietary supplementation with lactobacilli improves growth rate [4,5], enhances the immune response [6], and reduces gastrointestinal tract infections in pigs [7]. *Lactobacillus* spp. suitable for use as probiotics must have several essential features, including tolerance to acid and bile salt, adherence to intestinal epithelial cells, inhibition of pathogenic bacterial growth, non-pathogenicity, and no antimicrobial-resistance genes [8].

Since there are many *Lactobacillus* strains, each animal’s ideal probiotic strain should be isolated and characterized from the homologous host. Probiotic bacteria isolated from one animal species were best able to colonize the homologous animal intestinal tract and showed the best growth-stimulating performance in the homologous host [9]. Screens of probiotic bacteria usually focus on commercially bred pigs raised in the farm system, and there have been few reports of *Lactobacillus* isolation from native pigs raised on food scraps (backyard pig). Since backyards pigs are fed on leftovers, they are less likely to be exposed to antimicrobials

This study aimed to evaluate the *in vitro* probiotic properties of *Lactobacillus* isolated from indigenous swine manure.

## Materials and Methods

### Ethical approval

This study required no ethical approval as the collection of samples was from indigenous swine manure. All the other experiments were performed *in vitro*.

### Study period and location

Samples were collected from February 2019 to April 2019, and the study was performed from February 2019 to January 2020, at the Faculty of Science, Thaksin University (Phatthalung Campus), Thailand.

### Isolation of *Lactobacillus* from pig manure

A total of 30 individual fecal samples from healthy native pigs were collected immediately after excretion. All fecal specimens were collected in sterile plastic zip bags, immersed in iceboxes, and sent to the laboratory for immediate isolation. Approximately 2 g of each fecal sample was diluted ten-fold in sterile 0.85% NaCl, and then 10 mL of each 10^−3^-10^−5^ dilution was spread directly on de Man, Rogosa, Sharpe (MRS; HiMedia, India) agar supplemented with 0.01% (w/v) bromocresol purple. The plates were incubated anaerobically at 37°C for 24-48 h, and then at least five colonies surrounded by a yellow zone were selected and purified for further characterization. Colonies of Gram-positive, non-spore-forming bacilli, negative for catalase enzyme, and indole production were preserved at –80°C for further study.

### Antimicrobial activity against pathogenic bacteria

The inhibitory activity was investigated by the agar well diffusion method [10] against Enterohemorrhagic *E. coli* (EHEC, isolated strain), Enteropathogenic *E. coli* (EPEC, isolated strain), *Staphylococcus aureus* (ATCC 25923), *Klebsiella pneumoniae* (ATCC 700603), *Pseudomonas aeruginosa* (ATCC 27853), and *Salmonella* Typhimurium (extended-spectrum beta-lactamases [ESBL]-producing strain; isolated strain) [11]. In brief, all the selected bacteria were cultured in MRS broth (HiMedia: India) for 48 h, then cell-free supernatants (CFSs) were collected by centrifugation at 8000×*g* for 10 min and sterilized using a 0.2-mM sterile syringe filter membrane (Sartorius, USA). The supernatant was divided into three tubes. There was no need to adjust the pH in the first tube, and the second and third tubes were adjusted to pH 4.5 and 5.0 with 5 N NaOH and a pH meter (Sartorius, USA). The antimicrobial activity of the CFSs at different pH was investigated. The pathogenic indicator strain was adjusted to 0.5 McFarland standard (1.5×10^8^ cfu mL^−1^) (Den-1B, suspension turbidity detector, BioSan England) in 0.85% NaCl before spreading on tryptic soy agar (TSA) (HiMedia, India). Using a 6-mm Cork borer, holes were punched in the TSA plate, and 80-mL CFS was applied to each agar well. The TSA plate was incubated at 37°C for 24 h, and the inhibitory activity was observed by measuring the clarified zone around each agar well (measured in mm).

### Acid and bile salt tolerance

The preparation of the inoculum for acid and bile salt tolerance assays was performed as described by Ehrmann *et al*. [12]. All isolates were incubated anaerobically in MRS broth at 37°C for 48 h, and sub-cultured in MRS broth for 24 h, then adjusted to 0.5 McFarland in phosphate-buffered saline (PBS), pH 7.4.

For acid tolerance testing, 100 mL (0.5 McFarland) of each strain in MRS broth was adjusted to pH 2.0 and 3.0 with 1 M HCl and incubated anaerobically at 37°C for 0 and 3 h. Aliquots (100 mL) were spread on MRS agar and incubated at 37°C for 48 h. Survival was calculated as follows:

Survival percentage = (N_1_/N_0_) × 100

N_0_ = Initial inoculum quantity,

N_1_ = number of viable bacteria.

For the bile salts tolerance assay, a 100-mL, 0.5 McFarland aliquot of each strain was treated with 1% (w/v) bile salt solution (HiMedia, India) and incubated anaerobically at 37°C for 0 and 3 h. The survival rate in 1% bile salt solution was performed as described for the acid tolerance test.

### Biofilm formation assay

Biofilm formation was assessed in triplicate in 96-well microtiter plates, adapted from a previously described method [13]. Briefly, inoculum strains were cultured in MRS agar for 48 h and adjusted to 0.5 McFarland standards in 0.85% NaCl. A 180-μL aliquot of MRS broth supplemented with 5% (w/v) glucose per well was inoculated with 20-mL inoculum, then anaerobically incubated at 37°C for 36 h. The MRS broth was discarded from each well, washed twice with 200-mL sterile distilled water, and washed twice with 200-mL 0.85% NaCl. The plate was dried at room temperature for about 30 h before staining with 0.05% (v/v) crystal violet (HiMedia, India) for 50 min. The staining solution was removed, and each well was washed twice with 200-mL distilled water. The plate was dried at room temperature for 30 min, and then 200 μL 95% ethanol was added and transferred to a new 96-well plate. Optical density was measured at 600 nm. *P. aerug*inosa (ATCC 27853) and *E. coli* DH5-α were used as positive and negative controls for biofilm production.

### Hydrophobicity

The hydrophobicity assay was adapted from a published method [14]. In brief, the isolated strains were cultured in MRS broth for 24 h, and then the cells were collected by centrifugation at 8000×*g* for 10 min and washed twice with PBS (pH 7.4). The cell pellet was resuspended and adjusted to OD_600_ 0.6 in PBS (designed as A0), then a 3-mL aliquot was added to 1-mL xylene (C_6_H_4_(CH_3_)_2_), shaken vigorously for 2 min using a vortex mixer, and left to stand at room temperature for 15 min to allow separation of the aqueous phase. The reduction in absorbance was measured by spectrophotometry at 600 nm (designated A1). The hydrophobicity percentage of each isolate was calculated as follows:

% hydrophobicity = [(A_0_−A_1_)/A_0_]×100.

### Evaluation of autoaggregation and coaggregation

Autoaggregation and coaggregation testing were based on a published method of Dias *et al*. [15]. Briefly, the bacteria were cultured anaerobically in MRS broth at 37°C for 48 h, then washed twice with PBS (pH 7.4) and adjusted to OD_600_ 0.6. A 5-mL aliquot was shaken vigorously for 2 min using a vortex mixer and incubated for 4 h at room temperature. A 10-mL aliquot of the upper suspension was gently transferred to a 96-well plate containing 190-mL PBS, and the reduction in absorbance was measured at 600 nm. The autoaggregation percentage of each isolate was calculated as follows:

1 − (OD of upper suspension/OD of Total bacterial suspension)×100.

The coaggregation assay was performed with pathogenic bacteria, *S*. Typhimurium, EHEC, and EPEC, using an equal volume of each isolated strain and pathogenic strain adjusted to OD_600_ 0.6. Absorbance at 600 nm was measured again after 4 h incubation, and the coaggregation percentage was calculated as follows:

[{(A_pat_+A_probio_)/2-A_mix_}/(A_pat_+A_probio_)/2)]×100

A_pat_ and A_probio_ represent absorbance of the controls

A_mix_ represents the absorbance of the mixed bacteria.

### Adhesion to Caco-2 cells

The adhesion assay was performed as described by Sh *et al*. [16]. In brief, Caco-2 human epithelial colorectal adenocarcinoma cells (1.2×10^5^ cells mL^−1^) were cultured in 24-well plates with Dulbecco’s modified Eagles’ minimal essential medium (DMEM) supplemented with 10% (v/v) fetal bovine serum. Cells were incubated at 37°C in a CO_2_ incubator to confluence. The cells were washed twice with PBS (pH 7.4) after adding 1 mL of the isolated bacteria (0.5 McFarland standards) in serum-free DMEM and incubated at 37°C in a CO_2_ incubator for 90 min. The media was removed, and the wells were washed 3 times with PBS. After washing, 1 mL of 0.05% (v/v) Triton X-100 was added to each well and incubated at room temperature for 10 min. The Triton X-100 was diluted 10-fold with sterile normal saline, spread onto MRS agar, and incubated anaerobically at 37°C for 48 h. Colonies were counted, and the percentage of adhesion ability was calculated as follows:

% adhesion ability = (Number of bacteria attached to cells/total cells number)×100.

### Hemolytic activity and antimicrobial susceptibility testing

One colony of each isolated strain was picked from the MRS agar and re-streaked onto Columbia blood agar (5% human blood), then incubated anaerobically at 37°C for 48 h and observed for hemolysis. All isolates were tested with eight antimicrobial drugs (ampicillin, cephalothin, chloramphenicol, erythromycin, vancomycin, norfloxacin, streptomycin, and tetracycline) (HiMedia), using the disc diffusion technique recommended by the Clinical and Laboratory Standards Institute [17].

### Identification of the isolates bacteria by *16S rRNA* gene sequencing

Bacterial DNA extraction was performed using a kit (G-spin Genomic DNA Extraction Kit, iNtRON Biotechnology, Korea). Primers 27F (5Fmersnologynl,m beta-lactam and 1492R (592Rrsnologynl,m beta-lactams were used to amplify the *16S rRNA* gene in a thermocycler (MULTIGENE mini, Labnet, USA) in 50-μL platinum TM hot start Polymerase chain reaction (PCR) master mix (Thermo Fisher). Cycling conditions were as follows: Initial activation at 95°C for 10 min; 30 cycles of denaturation at 95°C for 1 min, annealing at 50°C for 1 min, and extension at 72°C for 90 s. The final cycle was 72°C for 5 min. The 1466-bp products were analyzed by agarose gel electrophoresis, purified with a kit (NucleoSpin Gel and PCR Clean-up Kit, Macherey-Nagel, England), and sequenced (Macrogen, Korea). Sequences were aligned using MEGA X software (www.megasoftware.net) [18] and compared by BLAST to representative sequences in GenBank. The phylogenetic tree was constructed based on the maximum likelihood method.

### Statistical analysis

The results on acid resistance, bile salt resistance, autoaggregation, coaggregation, and hydrophobicity were analyzed by one-way analysis of variance. Biofilm production in test and control groups was analyzed by t-test. All statistical tests were performed with Prism V.5 software (GraphPad Software, San Diego, CA, USA).

## Results

### Isolation of *Lactobacillus* from pig feces

In this study, a total of 314 isolates of lactic acid bacteria were isolated from 30 samples of individual native pig manure. The preliminary identification showed that all strains were Gram-positive, rod-shaped, non-motile, non-spore-forming, and negative for acid production, catalase, and indole production, a property of *Lactobacillus* spp.

### Antimicrobial activity against pathogenic bacteria

Of 314 isolates, 288 (91.72%) inhibited the growth of *K. pneumoniae*, 277 (88.21%) inhibited the growth of *S. aureus*, 264 (84.08%) inhibited the growth of EHEC, 225 (81.21%) inhibited the growth of EPEC, 242 (77.07%) inhibited the growth of *P. aeruginosa*, and 212 (67.52%) inhibited the growth of *S*. Typhimurium. We identified 78 isolates that inhibited the growth of all pathogenic strains tested, and these were selected for further characterization. Isolates were selected if they inhibited the growth of 6 pathogenic bacteria (inhibition zone at least 12 mm) and exhibited >50% survival in pH 3.0 and 1% bile salts: 17 isolates met these criteria (Table-1). To determine whether the inhibitory effect resulted from acid or bacteriocin formation, we took cell-free media from each isolate, adjusted the pH to 4.5 and 5.0, and re-tested for inhibition of EHEC, *S. aureus*, and *S*. Typhimurium again. The results showed that the antibacterial efficacy of all 17 isolates decreased with increased pH, suggesting that the pathogenic bacteria’s inhibition was due to the generated acid (Table-2).

**Table-1:** The 17 isolates of lactic acid bacteria that exhibited antibacterial activity and well tolerate to acid and bile salt.

Isolates	Inhibition zone (mm)*						Acid tolerance (%)	Bile salt tolerance (%)

	EHEC	EPEC	*S. aureus*	* K. pneumoniae*	* P. aeruginosa*	* S.* Typhimurium	pH 3.0	1% Bile salt
B1	30	18	15	19	14	12	88	100
B3	30	22	12	19	23	24	70.45	76
B4	25	16	16	16	20	19	100	58.90
B5	26	16	18	13	22	21	89.65	60.56
B8	25	17	18	18	19	22	100	100
B10	25	15	12	18	20	18	100	60
B11	25	18	15	17	21	18	53.85	100
B16	26	16	16	18	24	17	81.81	80
B17	30	18	16	18	23	19	100	100
B21	32	20	13	25	24	24	100	58.62
B27	32	18	17	17	23	17	100	80.64
B87	14	14	15	16	15	14	67.33	100
B88	14	16	21	20	14	12	100	100
B93	15	17	16	20	14	15	100	86.45
B126	20	17	20	21	16	22	100	80.64
B144	16	15	20	13	13	17	80	61
B172	23	20	30	15	21	24	80	71

* Cork borer: 6 mm in diameter. EHEC and EPEC: (isolated strain), *S. aureus* (ATCC 259), *K. pneumoniae* (ATCC 700603), *P. aeruginosa* (ATCC 27853), *S.* Typhimurium (isolated strain). EHEC=Enterohemorrhagic *Escherichia* coli, EPEC=Enteropathogenic *Escherichia coli*, *S. aureus*=*Staphylococcus aureus, K. pneumonia*=*Klebsiella pneumonia, P. aeruginosa*=*Pseudomonas aeruginosa*, *S.* Typhimurium=*Salmonella* Typhimurium

**Table-2:** The antimicrobial activity of supernatants under various pH values.

Isolate	pH of supernatant	EHEC			*S.* aureus			*S.* Typhimurium		
		
		Clear zone (mm)*			Clear zone (mm)			Clear zone (mm)		
		
		Original	pH 4.5	pH 5.0	Original	pH 4.5	pH 5.0	Original	pH 4.5	pH 5.0
B1	3.58	30	27	20	15	9	7	12	7	6
B3	3.38	30	27	22	12	8	6	24	13	7
B4	3.77	25	22	18	16	8	6	19	14	6
B5	3.67	26	21	19	18	9	6	21	17	9
B8	3.76	25	19	16	18	10	7	22	15	9
B10	3.88	25	19	14	12	7	6	18	9	7
B11	3.73	25	20	14	15	8	6	18	10	7
B16	3.76	26	20	13	16	8	6	17	10	6
B17	3.45	30	24	14	16	8	6	19	11	7
B21	3.44	32	24	13	13	7	6	24	14	8
B27	3.56	32	24	15	17	8	6	17	10	8
B87	4.01	14	9	7	15	8	6	14	7	7
B88	3.98	14	8	6	21	9	7	12	7	6
B93	3.85	15	8	6	16	8	6	15	6	6
B126	3.78	20	16	9	20	10	7	22	14	8
B144	3.93	16	10	7	20	10	7	17	6	6
B172	3.77	23	18	8	30	16	9	24	16	9

* Cork borer: 6 mm in diameter. EHEC: (isolated strain), *S. aureus* (ATCC 259), *S.* Typhimurium (isolated strain). EHEC=Enterohemorrhagic *Escherichia* coli, EPEC=Enteropathogenic *Escherichia coli, S. aureus*=*Staphylococcus aureus, S.* Typhimurium=*Salmonella* Typhimurium

### Acid and bile salt tolerance

The 78 isolates that exhibited activities against all pathogenic strains were selected for acid and bile salt tolerance assays. None of the isolates could survive at pH 2.0, but all 78 isolates survived to varying degrees when exposed to pH 3.0. In the 1% bile salt tolerance test, all 78 isolates survived at various rates. Therefore, to obtain high potential probiotic lactobacilli, isolates that showed >50% survival in an environment with pH 3.0 and 1% bile salt solution were selected for further characterization. We identified only 17 isolates (B1, B3, B4, B5, B8, B10, B11, B16, B17, B21, B27, B87, B88, B93, B126, B144, and B172) with a survival rate of >50% in an environment with pH 3.0 and with 1% bile salt solution, and all inhibited all six pathogenic bacteria (Table-1).

### Biofilm formation

**Figure-1:**
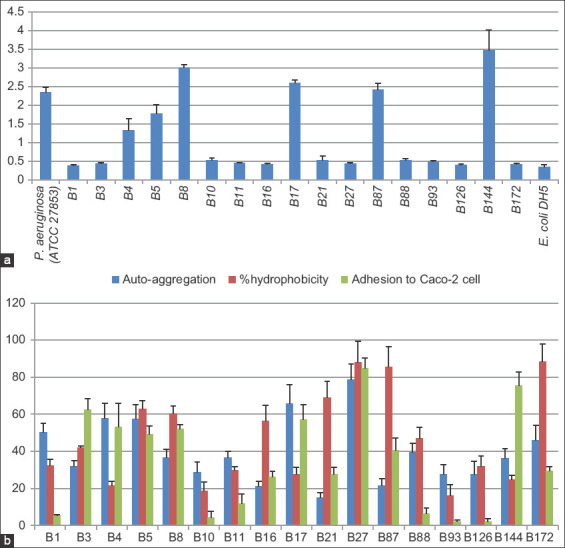
(a) Graph showing the biofilm production of the 17 isolates. The obtained value was shown from the average values of the three repeated experiment±standard deviation (SD). (b) Graph showing the percentage of auto-aggregation, hydrophobicity, and adhesion to Caco-2 cell. The obtained value was demonstrated from the average values of the three repeated experiment±SD.

Only six isolates (B4, B5, B8, B17, B87, and B144) produced more biofilm than the *E. coli* DH5-α-negative control, with statistical significance. Four of these isolates (B8, B17, B87, and B144) produced more biofilm than *P. aeruginosa*, which was used as a positive control, and two isolates (B144 and B8) produced significantly denser biofilms than *P. aeruginosa* (Figure-1a).

### Autoaggregation, hydrophobicity, and adhesion to Caco-2 cells

Only three isolates (B5, B8, and B27) revealed consistent results in autoaggregation, hydrophobicity, and Caco-2 cell adhesion. Isolate B27 exhibited a particularly strong ability to adhere to intestinal epithelial cells, with 88% hydrophobicity, 78.54% autoaggregation, and 84.66% adhesion to Caco-2 cells (Figure-1b). Considering the hydrophobicity results separately, there were seven isolates (B5, B8, B16, B27, B87, and B172) with a hydrophobicity index of >50% (Figure-1b). The autoaggregation assay showed clumping of all 17 isolates ranging from 19.08%-71.63%. Most showed an autoaggregation percentage of <50%. Only four isolates (B4, B5, B17, and B27) had an autoaggregation value of >50%, with isolates B27 and B172 being the highest (Figure-1b). Only seven isolates (B3, B4, B5, B8, B17, B27, and B144) showed >50% adhesion to Caco-2 cells (Figure-1b).

### Coaggregation with pathogenic bacteria

**Figure-2:**
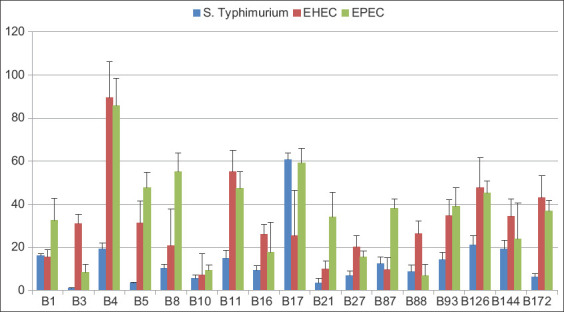
Graph showing the co-aggregation of the 17 isolates together with *Salmonella* Typhimurium, Enterohemorrhagic *Escherichia* coli, and Enteropathogenic *Escherichia coli*. The obtained value was demonstrated from the average values of the three repeated experiment±standard deviation.

We selected *S*. Typhimurium, EHEC, and EPEC to represent enteric pathogens for the coaggregation assay. All 17 isolates aggregated with EHEC and EPEC more than with *S*. Typhimurium, most with an aggregation percentage of >50% with *E. coli* especially. Isolate B4 bound EHEC and EPEC at 71.96% and 91.44%, respectively (Figure-2). However, only isolate B17 aggregated with *S*. Typhimurium at an efficiency of >50 % (Figure-2).

### Antimicrobial susceptibility and hemolysis

Antimicrobial susceptibility testing showed that all isolates were susceptible to cephalothin, chloramphenicol, erythromycin, streptomycin, and ampicillin, but only one isolate (B27, 5.88%) was resistant to tetracycline. It was notable that only 17.64% (B1, B3, and B5) were sensitive to vancomycin, and 11.76% (B1 and B5) were sensitive to norfloxacin. Three isolates (B1, B3, and B10) exhibited partial or incomplete lysis of red blood cells (a-hemolysis) with a 1-mm partial or slightly green clear zone around the bacterial colonies. In contrast, the remaining 14 isolates (B4, B5, B8, B17, B11, B16, B21, B27, B87, B88, B93, B126, B144, and B172) exhibited non-hemolysis (γ-hemolysis) (Table-3).

**Table-3:** Antimicrobial susceptibility testing, hemolysis assay, and identification.

Isolate code	Antimicrobial susceptibility testing								Hemolysis type	Identification (% identity)* Accession No.	

	CEP	CHL	ERY	STR	AMP	TET	NOR	VAN			
B1	S	S	S	S	S	S	S	S	α	*L. plantarum* MW165836	(100)
B3	S	S	S	S	S	S	R	S	α	*L. plantarum* MW165837	(99.74)
B4	S	S	S	S	S	S	R	R	γ	*L. plantarum* MW165838	(99.74)
B5	S	S	S	S	S	S	S	S	γ	*L. plantarum* MW165839	(99.67)
B8	S	S	S	S	S	S	R	R	γ	*L. plantarum* MW165840	(98.67)
B10	S	S	S	S	S	S	R	R	α	*L. plantarum* MW165841	(99.60)
B11	S	S	S	S	S	S	R	R	γ	*L. plantarum* MW165842	(99.54)
B16	S	S	S	S	S	S	R	R	γ	*L. plantarum* MW165843	(100)
B17	S	S	S	S	S	S	R	R	γ	*L. plantarum* MW165844	(99.54)
B21	S	S	S	S	S	S	R	R	γ	*L. plantarum* MW165845	(99.30)
B27	S	S	S	S	S	R	R	R	γ	*L. salivarius* MW165846	(99.35)
B87	S	S	S	S	S	S	R	R	γ	*L. plantarum* MW165847	(99.67)
B88	S	S	S	S	S	S	R	R	γ	*L. plantarum* MW165848	(99.21)
B93	S	S	S	S	S	S	R	R	γ	*L. plantarum* MW165849	(99.21)
B126	S	S	S	S	S	S	R	R	γ	*L. plantarum* MW165851	(99.53)
B144	S	S	S	S	S	S	R	R	γ	*L. plantarum* MW165852	(99.00)
B172	S	S	S	S	S	S	R	R	γ	*L. plantarum* MW165850	(99.27)

CEP=Cephalothin, CHL=Chloramphenicol, ERY=Erythromycin, STR=Streptomycin, AMP=Ampicillin, VAN=Vancomycin, NOR=Norfloxacin, TET=Tetracycline. R=Resistant (zone diameter, ≤12.4 mm) S=Susceptible (zone diameter, ≥17.5). Erythromycin results based on R ≤13 mm; S ≥23 mm. Gentamycin results based on R ≤6 mm; S ≥10 mm. Vancomycin results based on R ≤12 mm; S ≥13 mm.

* All *Lactobacillus plantarum* were blast against *Lactobacillus plantarum* NR_042254.1, *Lactobacillus salivarius* was blast against *Lactobacillus salivarius* NR_028725.2

### Identification by *16S rRNA* gene sequencing

**Figure-3:**
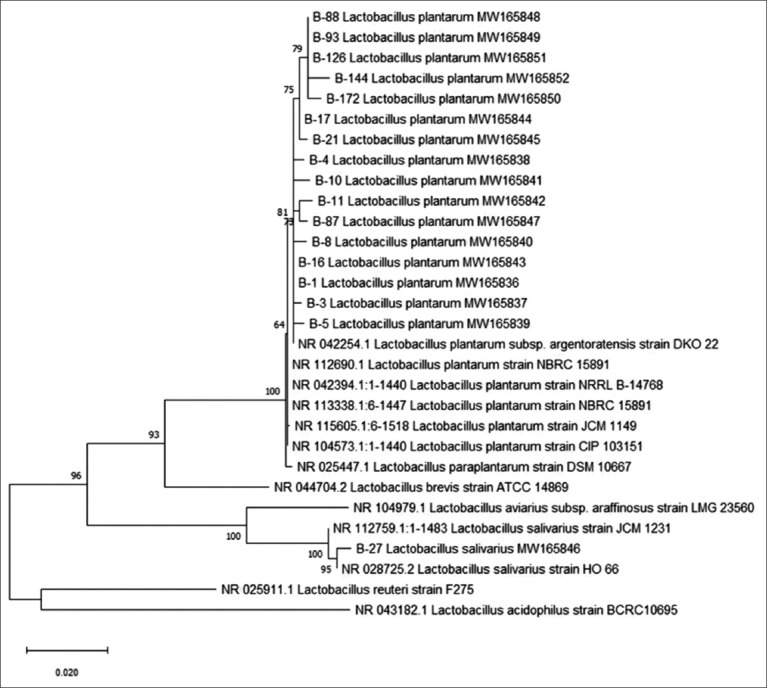
The relationship of *Lactobacillus* analyzes results and constructs a phylogenetic tree with the MEGA X program by the maximum likelihood method (1000 bootstrap).

All 17 isolates were characterized by *16S rRNA* gene sequencing, and a phylogenetic tree was constructed with the nucleotide sequences retrieved from GenBank. The analysis showed 16 of the isolates were *Lactobacillus plantarum*, with identities ranging from 98.69% to 100%, and one isolate (B27) shared 99.35% identity with *Lactobacillus salivarius*. The GenBank Accession numbers for the *16S rRNA* gene sequence of the 17 isolates are MW165836–MW165850 (Table-2 and Figure-3).

## Discussion

In this study, 314 isolates of *Lactobacillus* were isolated from the manure of 30 native pigs to evaluate their probiotic potential. Initially, we selected 78 isolates of lactic acid bacteria that could inhibit all six pathogenic bacteria: *E. coli* (EPEC and EHEC), *S. aureus*, *K. pneumoniae*, *P. aeruginosa*, and *S*. Typhimurium (ESBL-producing strain). These bacteria are a significant problem in the health of pigs and may affect human health [19,20]. This study shows that bacteria isolated from healthy native pigs are highly effective in inhibiting pathogenic bacteria. The previous study found that *Lactobacillus* isolated from commercial pigs, native pigs, and wild boars are highly efficient inhibitors of some pathogenic bacteria [21]. There are disadvantages to isolating *Lactobacillus* from commercially farmed pigs because they are typically given probiotic *Lactobacillus* in their feed, making it unlikely that new strains will be identified in these populations.

Antibacterial efficacy decreased when the pH of the CFS was neutralized, indicating that the ability to inhibit pathogens is mostly a result of acid production. This study is consistent with the previous studies showing that *Lactobacillus* can inhibit Gram-positive and Gram-negative pathogens, especially *E. coli* and *S. aureus*, but are less effective against *S*. Typhimurium, which is more resistant to acids than other enteric pathogens [22,23].

The previous study isolated *Lactobacillus* from farm pig manure and small intestines obtained in slaughterhouses and showed these strains are less tolerant of acids and bile salts than the isolates obtained in our study [24]. This difference indicates the importance of feeding with kitchen leftovers; backyard native pigs eat regularly, requiring digestion with more acid, and bile salts. The acid and bile salt-resistant bacteria therefore survive and can be more frequently detected in native backyard pigs. Backyard pigs also have a higher chance of exposure to pathogens. These conditions create more opportunities to find a group of lactobacilli that can inhibit pathogenic bacteria.

Acid and salt resistances are essential qualities that make certain probiotic bacteria survive extreme conditions and colonize the digestive tract. We tested the isolates with 1% bile salt, a higher concentration than is found in the intestines of pigs [22,25]. We found 17 isolates that exhibited >50% survival under these conditions. Nevertheless, none survived at pH 2.0. Therefore, we adjusted to pH 3.0, the stomach pH range of pigs [26-28], and found >50% survival. Acid tolerance is driven by the F0F1-ATPase mechanism, which is a multi-unit enzyme consisting of catalysts (F1) a, b, g, d, and e for ATP hydrolysis, and the integral membrane (F0) a, b, and c subunits, which act as channels for proton transport [29]. These mechanisms mediate bacterial respiration through proton transport, suggesting that F0F1-ATPase can increase the cell’s pH under low pH conditions by regulating gene expression. In contrast, bile salt tolerance results from bile efflux and bile hydrolysis [30]. Isolates that can withstand such conditions may possess more effective enzymes.

Assays to identify the ability of bacteria to colonize the gastrointestinal tract efficiently include autoaggregation, hydrophobicity, and adhesion to epithelium cells (Caco-2). We employed all three methods to identify isolates with concordant properties. Only three isolates (B5, B8, and B27) exhibited consistent results between the three assays. Coaggregation between the same or different bacterial strains is essential to biofilm formation [31]. However, only isolates B5 and B8 exhibited *in vitro* adhesion properties and produced biofilm polysaccharides at high levels.

In contrast, isolate B27 showed strong evidence of adhering to intestinal epithelial cells but expressed low levels of biofilm polysaccharides, indicating that this is not the only mechanism involved in biofilm formation. A recent study suggested that *L. plantarum* produces a surface protein that plays an important role in adherence to the intestinal epithelium. Sodium dodecyl sulfate and mass spectrometry analyses indicated that the surface protein is 100% homologous to glyceraldehyde-3-phosphate dehydrogenase (GAPDH). Moreover, blocking of GAPDH by anti-GAPDH significantly decreased adherence to intestinal epithelial cells [32]. Likewise, *L*. *sarivarius* expresses an S-layer choline-binding protein A (CbpA) involved in adherence to the human colorectal adenocarcinoma cell line HT-29. Blockade or deletion of CbpA significantly decreased the ability of *L. salivarius* to adhere to epithelial cells [33]. These alternative models of adhesion may account for the fact that isolate B27 produces low levels of biofilm polysaccharides but can efficiently adhere to the intestinal wall cells.

Antimicrobial resistance and red blood cell hemolysis are common considerations for screening probiotic bacteria properties. In this study, most isolates were sensitive to all antimicrobial agents, and only one isolate (B27) was resistant to tetracycline. However, we found that most *Lactobacillus* isolates were resistant to vancomycin and norfloxacin, and only three (B1, B3, and B5) were susceptible to vancomycin, and two (B1, B5) were susceptible to norfloxacin. Vancomycin resistance is not uncommon in *Lactobacillus*. It is well-known that the terminal D-alanyl-D-alanine residue in the pentapeptide crosslink of several *Lactobacillus* species is substituted with D-alanyl-D-lactate, to which vancomycin binds with 1000-fold lower affinity [34]. Substitution of D-alanyl-D-alanine with D-alanyl-D-lactate is mediated by Ddl ligase, an essential enzyme in *Lactobacillus* peptidoglycan synthesis. Heterologous expression of Ddl ligase in *L. plantarum* reportedly increases sensitivity to vancomycin [35]. The lactobacilli are also naturally resistant to quinolones (ciprofloxacin, norfloxacin, and nalidixic acid) through an unknown resistance mechanism; however, we cannot explain why isolates B1 and B5 are sensitive to norfloxacin. This study found that only 5% of *Lactobacillus* was resistant to tetracycline, in contrast to the previous studies that showed 80% of the *Lactobacillus* isolated from commercial farm piglets was resistant to tetracycline [36]. *Lactobacillus* strains isolated from weaning commercial farm piglets are more frequently resistant to other antimicrobial drugs, suggesting that researchers have a better chance of isolating antimicrobial-susceptible *Lactobacillus* from native backyard pigs. In addition to hemolysis, almost all isolates exhibited γ, hemolysis (except isolates B1, B3, and B10), indicating that most of them are relatively safe to use as animal probiotics. Although the probiotic bacteria must be safe and have zero risks, it is difficult to find an entirely safe bacterium. Some *Lactobacillus* species, *Leuconostoc*, and *Pediococcus* may show incomplete or partial hemolysis (γ-hemolysis) [37,38].

A preliminary classification by *16s rRNA* gene analysis found that *L. plantarum* was the most common isolate, and B27 was identified as *L. salivarius*, but this does not mean that there is no bacterial diversity in native pigs. Our sequential screening process may have enriched for *L. Plantarum*. The prevalence of *Lactobacillus* species varies depending on location in the intestinal tract, age, health status, and diet. *L. plantarum* and *L. fermentum* are prevalent in swine manure [39-41]. It may be necessary to understand that the *16S rRNA* gene nucleotide sequence may not distinguish all *Lactobacillus* species because this gene is highly conserved, especially in *L. plantarum*, *L. paraplantarum*, and *L. pentosus*. Differentiating these species may require additional techniques, including the PCR-ARDA and specific gene amplification [42]. Although most isolates are *L. plantarum*, each represented a different strain according to their other characteristics. We thus cannot conclude that they are all *L. plantarum*.

## Conclusion

An *in vitro* screen of the probiotic properties of *Lactobacillus* isolated from pig manure yielded 17 strains from a total of 314 isolates that exhibited favorable probiotic and safety characteristics. Sixteen of these isolates were primarily identified as *L. plantarum*, and one isolate was identified as *L. salivarius*. Each isolate has different probiotic strengths and weaknesses. Therefore, their development and application as a novel swine feed supplement will require *in vivo* investigations to optimize the ideal probiotic cocktail.

## Authors’ Contributions

ML: Designed, planned, and supervised the study. CT: Performed all the experiments and collected the data. CT, AS, and ML: Analyzed the results. CT wrote the manuscript. ML and AS edited and finalized the manuscript. All authors read and approved the final manuscript
